# The role of cardiac rehabilitation using exercise to decrease natriuretic peptide levels in non-surgical patients: a systematic review

**DOI:** 10.1186/s13741-019-0124-0

**Published:** 2019-11-18

**Authors:** Christella S. Alphonsus, Pooveshni Govender, Reitze N. Rodseth, Bruce M. Biccard

**Affiliations:** 1 University of Cape Town, Cape Town, Western Cape South Africa; 2 D23, Groote Schuur Hospital, Anzio Road, Observatory, Cape Town, Western Cape 7925 South Africa; 3 The Royal Papworth Hospital, Cambridgeshire, UK; 4 University of KwaZulu-Natal, Durban, KwaZulu-Natal South Africa

**Keywords:** Cardiac morbidity, Preoperative factors, Myocardial ischaemia

## Abstract

Exercise is recommended in patients with cardiac failure. In the perioperative patient, exercise is also gaining popularity as a form of prehabilitation. In this meta-analysis, we examine if exercise is able to reduce natriuretic peptide levels. Natriuretic peptide (NP) has strong prognostic ability in identifying patients who will develop adverse postoperative cardiovascular outcomes. The protocol was registered with PROSPERO (CRD42017051468). The database search included MEDLINE (PubMed), CINAHL (EBSCO host), EMBASE (EBSCO host), ProQuest, Web of Science, and Cochrane database. The primary outcomes were to determine whether exercise therapy was effective in reducing NP levels as compared to control group, the shortest time period required to reduce NP levels after exercise therapy, and whether reducing NP levels decreased morbidity and mortality. Full texts of 16 trials were retrieved for this review. Exercise therapy showed a significant reduction in natriuretic peptide levels between the intervention and control groups (SMD − 0.45, 95% CI − 0.88 to − 0.03) with significant heterogeneity between the included trials. This was also shown in the within a 12-week period.

## Introduction

Historically, exercise was commonly avoided in patients with heart failure. This has changed dramatically over the past 30 years with recommendations from international organisations such as the European Society of Cardiology and American College of Cardiology Foundation/American Heart Association for the use of exercise training to improve exercise tolerance and reduce morbidity and mortality (Cattadori et al. 2018).

Exercise is now considered part of preoperative rehabilitation, also known as prehabilitation, for patients presenting for surgery. This is based on the philosophy that improving functional capacity may improve the patient’s ability to withstand the surgical stress response and thereby improve postoperative outcomes. Outcomes such as hospital length of stay, postoperative pulmonary complications, and quality of life have been improved with this approach (Drudi et al. 2019).

There is currently no consensus on the type and duration of exercise needed to improve outcome in surgical patients (Vermillion et al. 2018) and non-surgical patients (Smart and Steele 2010). Furthermore, B-type natriuretic peptide (BNP) testing has been recommended to identify patients at high risk of perioperative cardiovascular events (Duceppe et al. 2017), yet there remains limited data on the efficacy of exercise to decrease B-type natriuretic peptides (Smart and Steele 2010) and decrease subsequent cardiovascular events.

The objective of this systematic review of clinical trials was to determine whether in adult, medical patients with cardiac failure, exercise therapy was able to decrease natriuretic peptide (NP) levels and whether this was associated with improved cardiovascular outcomes.

## Methods

### Protocol and registration

The protocol was registered with PROSPERO (CRD42017051468). The Preferred Reporting Items for Systematic reviews and Meta-Analysis (PRISMA) guidelines were adhered to (Moher et al. 2009). This protocol included a meta-analysis included on the effect of medical therapy on NP levels, which is presented in the accompanying paper (Alphonsus et al. 2019).

### Eligibility criteria

The inclusion criteria for this systematic review have been described in the systematic review on natriuretic peptide-directed medical therapy which included exercise therapy trials.

In this systematic review, we report prospective randomised clinical trials of adult medical patients who were randomised to exercise as part of cardiac rehabilitation, where the subsequent changes in natriuretic peptide levels are reported. We excluded (i) trials that monitored natriuretic peptides for prognostic or diagnostic purposes, without a strategy to lower natriuretic peptide levels; (ii) reviews of natriuretic peptide or biomarker physiology; and (iii) trials reporting natriuretic peptides in patients with acute myocardial infarction, pulmonary hypertension, cardiac resynchronisation therapy, and left ventricular assist devices.

### Information sources, search, and study selection

Three searches were conducted using search terms ‘brain natriuretic peptide’ AND ‘treatment’, ‘brain natriuretic peptide’ AND ‘heart failure’, and ‘brain natriuretic peptide’ AND ‘exercise’. The following databases were accessed: MEDLINE (PubMed), CINAHL (EBSCO host), EMBASE (EBSCO host), ProQuest, Web of Science, and Cochrane database. There were no filters used for year of publication or language. Non-English titles were not excluded. An example of the search is shown in Additional file 1. The initial search was conducted on 22 December 2016 and updated on 4 March 2018.

### Data collection process

Titles were screened for potential inclusion by CA and PG. Abstracts of potential papers identified through the title search were then screened using inclusion and exclusion criteria by CA and PG. The full texts of potential trials were then extracted for full text review and analysis. Reference lists were searched for additional papers that could be included in this review. Data extraction was done by one author (CA) and then checked by a co-author (BB). When required data was not presented in the publication, the authors were contacted for these data.

### Data items

We extracted data on the NP reduction at the end of the exercise trials. Data on the patient characteristics, the type of exercise intervention, the physical activity in the control group, and the mortality and morbidity in the trials was also extracted.

### Outcomes

The primary outcomes for this review were to determine (i) whether exercise therapy was effective in reducing NP levels as compared to control group, (ii) the shortest time period required to reduce NP levels after exercise therapy, and (iii) whether reducing NP levels decreased morbidity and mortality. The secondary outcome was to determine which specific exercise regimens were more effective in reducing NP levels.

### Risk of bias in individual studies

Assessment of bias in the studies was conducted by CA and verified by BB following discussion. The Cochrane Collaboration risk of bias tool was used and assessed selection bias, concealment bias, performance bias, detection bias, attrition bias, and other biases. Studies were assessed as having low, unclear, or high risk of bias.

### Summary measures and synthesis of results

Statistical analyses were conducted using Review Manager Version 5.3 (Copenhagen: The Nordic Cochrane Centre, The Cochrane Collaboration, 2014). For NP reduction, we tabulated the absolute NP change. NP levels which were reported as median and interquartile range (IQR) were converted to mean and SD (Wan et al. 2014). As the included trials used either BNP or NT-proBNP to monitor therapeutic response, we made use of standardised mean difference (SMD) for our meta-analysis. SMD addresses the difference in the effect size for an intervention when the units of measurement differ between trials, e.g. use of BNP or NT-proBNP. The SMD is the difference between groups in mean end point divided by the SD of the control group (or pooled SD of the treatment and control groups) (Guyatt et al. 2015). These data are presented as a forest plot. Random effects models were used where the *I*^2^ statistic > 25% (representing significant heterogeneity); otherwise, a fixed effects model was used.

### Risk of bias across studies

Risk of publication bias across studies was assessed with funnel plots for NP reduction.

## Results

### Study selection

**Fig. 1:**
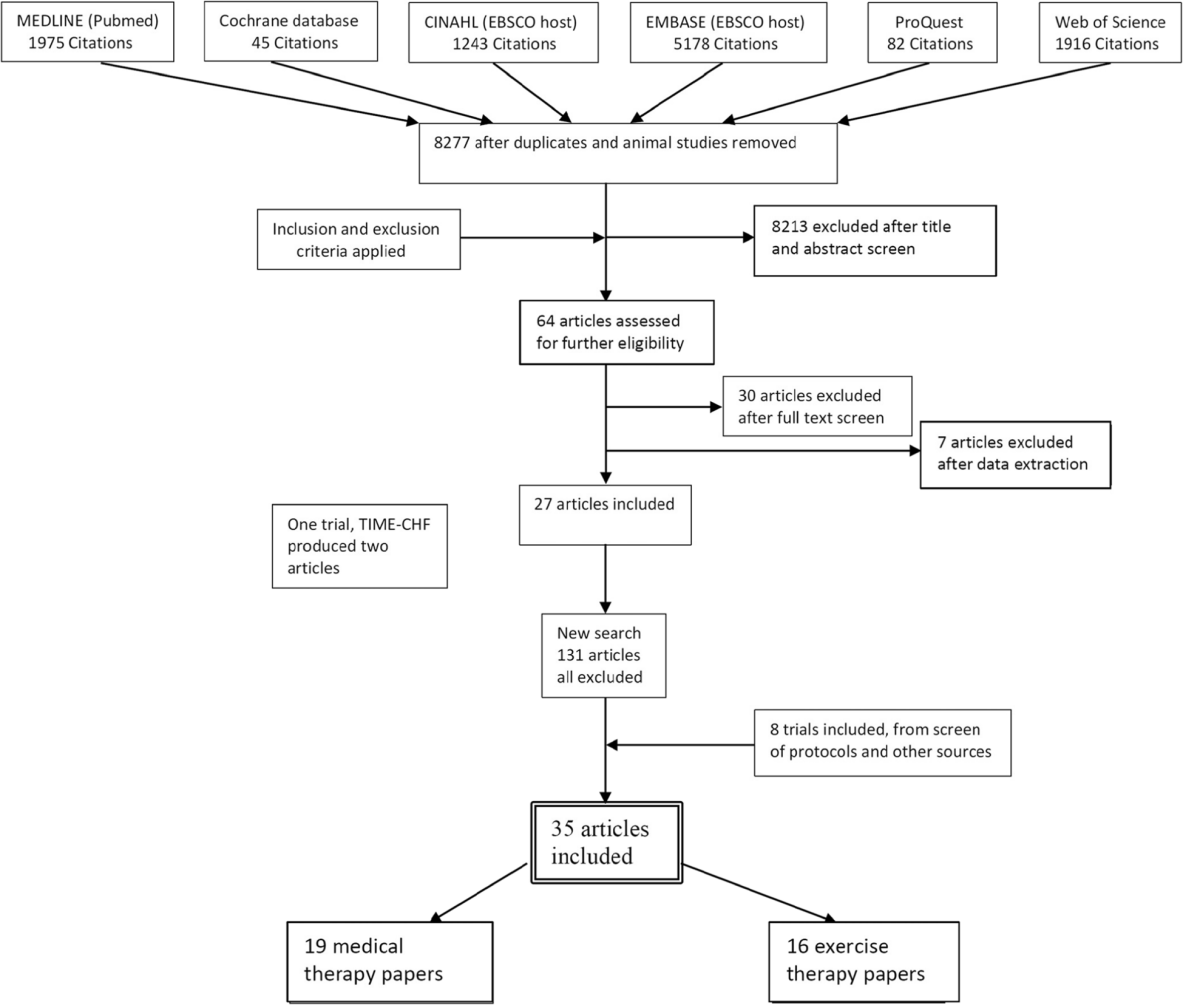
PRISMA flow diagram

After the initial search, 64 articles were reviewed for potential inclusion. Twenty-six trials (27 publications) were selected and 8 trials added from references, of which 18 were trials of medical therapy interventions and 16 trials were of an exercise intervention (Fig. 1).

We evaluated 2 previous systematic reviews using the AMSTAR format (Additional file 2).

### Study characteristics of included studies

Cardiac rehabilitation exercise trials were conducted in adult patients 18 years and older, in an outpatient setting (Table 1). The included cardiac rehabilitation exercise trials all included some form of aerobic exercise, either walking, bicycle, or treadmill. Trials that involved interval training were considered as a separate subgroup for analysis (Aksoy et al. 2015; Nilsson et al. 2010; Stevens et al. 2015). Three trials included resistance training in addition to aerobic training in the intervention group (Gary et al. 2011; Jonsdottir et al. 2006; Malfatto et al. 2009; Norman et al. 2012). Most trials ran for up to 12 weeks, one trial for 20 weeks (Jonsdottir et al. 2006), and another for 24 weeks (Norman et al. 2012). Most trials were small, with a maximum of 40 patients in each arm, with the exception of 1 large trial (HF-ACTION) which included 477 patients (Ahmad et al. 2014). Most trials included patients with an ejection fraction (EF) < 40%, two trials had patients with EF 40–49% (Guazzi et al. 2012; Parrinello et al. 2010), and three trials had patients with mixed categories of heart failure (Aksoy et al. 2015; Jonsdottir et al. 2006; Nilsson et al. 2010). The exercise intervention protocols were not individualised to the NP levels, but the NP response to the exercise intervention was reported in all the trials.

**Table 1:** Trial characteristics of cardiac rehabilitation exercise trials

Author, year	Patient characteristics	Type of NP	Baseline NP in intervention group (pg/ml)
Kobayashi et al. 2003	Stable NYHA II-III. EF < 40%	BNP	281 ± 92
Meyer et al. 2004	Stable NYHAII-III. EF ≤ 40%	NT-proBNP	1092 ± 980
Jonsdottir et al. 2006	Patients previously hospitalised in past 3 years for heart failure	BNP	173.2 ± 180.4
Maria Sarullo et al. 2006	Stable CHF. EF < 40%	NT-proBNP	3376 pg/ml ± 3133
Brubaker et al. 2009	CHF. EF ≤ 45%	BNP	176 ± 38
Malfatto et al. 2009	Chronic heart failure	BNP	293 ± 115
Parrinello et al. 2010	Stable NYHAII-III. EF ≤ 45%	BNP	205.2 ± 46.5
Gary et al. 2011	Stable NYHA II-III, stable on medical therapy. EF 15 to 40%	BNP	184.4_151.6
Guazzi et al. 2012	Stable NYHA class II or III, stable on medical therapy. EF ≤ 45%	NT-proBNP	1088.1 ± 447.1
Norman et al. 2012	Volunteers, NYHAII-IV, ≥ 21 years, LVEF ≤ 40%, on optimal medical therapy	BNP	1088.1 ± 447.1
Sandri et al. 2012	Stable CHF. EF < 40%	NT-proBNP	≤ 55 years, 1675 ± 354 ≥ 65 years, 1301 ± 261
Eleuteri et al. 2013	Stable NYHA II, stable on medical therapy. EF ≤ 40%	NT-proBNP	1570.7 ± 3125.8
Ahmad et al. 2014, HF-ACTION substudy	CHF patients with reduced left ventricular ejection fraction (< 35%)	NT-proBNP	960.6 ± 1114
Aksoy et al. 2015	NYHAII-III CHF on optimal medical therapy. EF 35 to 55%	NT-proBNP	Continuous aerobic exercise group 20.79 ± 12.8 Interval exercise group 24.00 ± 18.27

*NP* natriuretic peptide, *CHF* chronic heart failure, *EF* left ventricular ejection fraction, *BNP* B-type natriuretic peptide, *NT-proBNP* N-terminal pro-B-type natriuretic peptide, *NYHA* New York Heart Association

The exercise intervention group received supervised exercise training in all the trials, except two where the exercises were home-based after participants were given instructions (Eleuteri et al. 2013; Parrinello et al. 2010). The control group were given exercise information (except Brubaker (Brubaker et al. 2009)) but did not receive supervised exercise training.

### Risk of bias within studies and across studies

**Fig. 2:**
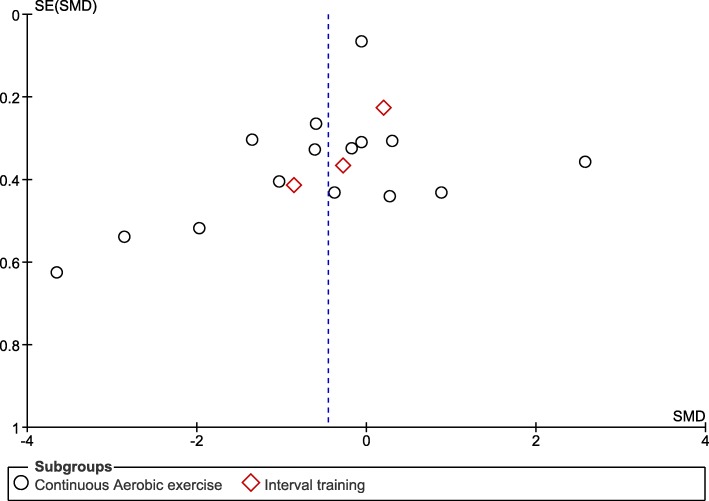
Funnel plot for standardised mean difference forest plot. SMD- standardised mean difference

The risk of bias of the included trials is shown in the Additional file 3: Figure S1 and Additional file 4: Figure S2. The random sequence generation was acceptable in three trials (Ahmad et al. 2014; Guazzi et al. 2012; Maria Sarullo et al. 2006). By virtue of the intervention (supervised exercise versus exercise recommendation), blinding of patients was impossible, and investigator blinding was poor. Outcome assessors were only blinded in two trials (Ahmad et al. 2014; Brubaker et al. 2009). The funnel plots for SMD (Fig. 2) did not suggest publication bias.

### Results of individual studies and synthesis of results

All trials presented data on NP levels at the end of the intervention period.

#### Is exercise therapy as effective in reducing NP levels as compared to control group?

**Fig. 3:**
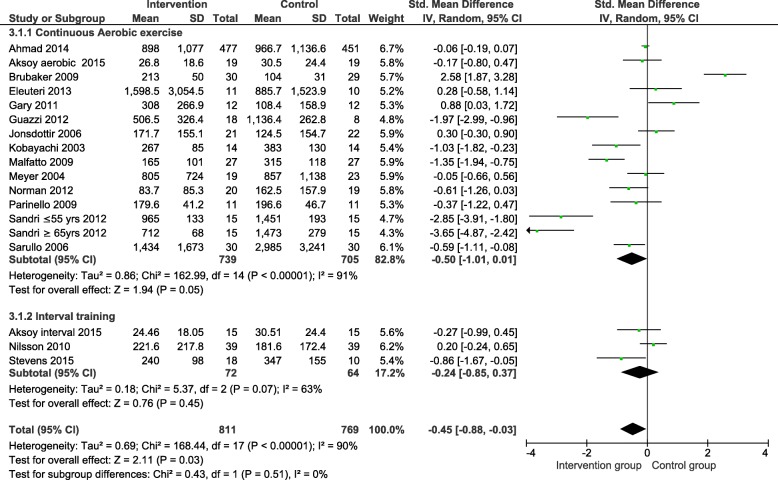
Standardised mean difference in natriuretic peptide levels in exercise therapy. SD-standard deviation; CI-confidence interval

The meta-analysis of the SMD in NP levels between the intervention and control group is shown in Fig. 3. The overall point estimate showed a significant reduction in NP levels between the intervention and control groups (SMD − 0.45, 95% CI − 0.88 to − 0.03) with significant heterogeneity between the included trials. Neither the continuous aerobic, nor the interval training subgroup showed a significant reduction in NP.

#### What is the shortest time period required to reduce NP levels after exercise therapy?

**Fig. 4:**
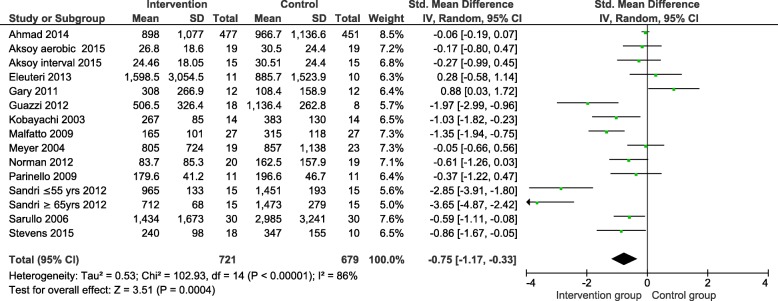
Standardised mean difference in natriuretic peptide levels in exercise therapy within 12 weeks. SD-standard deviation; CI-confidence interval

An analysis of the trials with a 12-week intervention period (the shortest exercise intervention period in the eligible trials) showed a significant NP reduction (SMD − 0.75, 95% CI − 1.17 to − 0.33) (Fig. 4).

#### Does reducing NP levels decreased morbidity and mortality?

Only four trials reported on mortality (Ahmad et al. 2014; Brubaker et al. 2009; Jonsdottir et al. 2006; Nilsson et al. 2010). HF-ACTION trial (Ahmad et al. 2014) reported 189 (16%) deaths in the intervention group and 198 (17%) deaths in the control group, HR 0.96 (0.79–1.17), *p* = 0.70, and a cardiovascular mortality at a median follow-up of 30 months of 131 (11%) deaths in the intervention group and 143 (12%) deaths in the control group, 0.92 (0.74–1.15), *p* = 0.47. Three other trials, Nilsson et al. (2010), Jónsdóttir et al. (2006), and Brubaker et al. (2009), reported mortality. Nilsson et al. reported one death in the control group, Jónsdóttir et al. two each in intervention and control groups, and Brubaker et al. one in each group. None of other trials reported mortality.

#### Secondary outcome: where specific exercise regimens were more effective in reducing NP levels?

Neither supervised continuous aerobic exercise or interval training was independently associated with a significant reduction in NP levels. A preferable exercise regimen therefore cannot be determined.

## Discussion

This meta-analysis examines exercise rehabilitation in adult patients with chronic heart failure in an outpatient setting. The principal finding was that exercise training can significantly decrease NP levels within 12 weeks in adult medical patients eligible for cardiac rehabilitation. The patients most likely to benefit from this intervention had stable chronic heart failure, with an EF which was predominantly < 45% (Table 1). However, significant heterogeneity exists between trials. Currently, there is insufficient data to determine whether this NP reduction is also associated with a survival benefit.

The characteristics of the exercise programmes were the following (Table 2). Almost all trials included exercise programmes that were conducted under direct supervision. All the programmes had an aerobic component and varied in exercise intensity, duration, and frequency. Few trials had interval training and resistance training. The exercise regimens were predominantly determined by patient-specific physiological parameters, e.g. AT, VT, and VO_2_ peak, and were thus individualised. The duration of the exercise programme was 12 weeks in most trials. These findings are applicable to patients with cardiac failure of varying severity but considered stable on medication. It is important to note that NP levels were not a criterion for inclusion in these trials, and hence, we do not know the baseline NP level necessary to determine eligibility for a supervised exercise programme.

**Table 2:** Characteristics of exercise programme in the intervention group

Author, year	Intervention	Time period	Control group activity
Kobayashi et al. 2003	Cycle 15 min. HR equivalent to VT. 2–3 times/week	12 weeks	Normal level of activity
Meyer et al. 2004	Cycle at for 45 min. 4 times/week	12 weeks	Not specified
Jonsdottir et al. 2006	Aerobic and resistance training. Cycle for 15 min, 50% of peak work load, and gradually increased then 20 min circuit training. 2 times/week	20 weeks	Normal level of activity
Maria Sarullo et al. 2006	Cycle 30 min 60–70% of peak VO2. 3 times/week	12 weeks	Normal level of activity
Brubaker et al. 2009	Cycle 3 times/week. Start at 40–50% of HRR then increased after 2 weeks to 60–70% HRR for 15–20 min	16 weeks	Normal level of activity
Malfatto et al. 2009	Cycle or treadmill 40 min HR to 60% of VO2 peak. 3 times/week	12 weeks	No training
Parrinello et al. 2010	Walking 30 min. 5 times/week	10 weeks	Normal level of activity
Gary et al. 2011	Walking and resistance. Target HR within prescribed range and rate of perceived exertion within 15	12 weeks	Stretching and flexibility exercises
Guazzi et al. 2012	Cycle ergometer 40 min. 60% HRR	12 weeks	Not specified
Norman et al. 2012	Aerobic exercise, 3 days a week, at 40%–70% heart rate reserve, based on the baseline CPET, or 11–14 on Borg scale for 30 min with 15 min warm-up and 15 min cool-down. Resistance training, 2 days a week, 8–10 exercises (upper and lower body) performed for one set of 10 to 15 repetitions, using weight machines, free weights or elastic bands. Supervised for first 3 weeks	24 weeks	No supervised exercise
Sandri et al. 2012)	Cycle 4 times/day. Workloads were adjusted to heart rate so that 70% of the symptom-limited maximum oxygen uptake was reached	4 weeks	Not specified
Eleuteri et al. 2013	Home based with calibrated cycle ergometer. 30 min 5 times/week. Power and HR corresponding to AT	12 weeks	Normal lifestyle activities
Ahmad et al. 2014, HF-ACTION substudy	Supervised 3 times per week: walking/treadmill/stationary cycling. Initial 15 to 30 min heart rate 60% of heart rate reserve and ramped up. At home: exercise adherence and amount formally measured	12 weeks	No supervised exercise
Aksoy et al. 2015	Supervised two groups, continuous up to 50% VO2 peak no change in intensity. Interval cycling with high and low intensity. 35 min 3 times/week	10 weeks	No exercise

*VO2* maximum oxygen consumption, *HR* heart rate, *HRR* heart rate reserve, *AT* aerobic training, *CPET* cardiopulmonary exercise training, *VT* ventilatory threshold

The strength of this meta-analysis is that it shows exercise training to be associated with a reduction in NP levels within 12 weeks from randomisation. There were no reports of morbidity associated with the supervised exercise programmes.

This review has some limitations. Firstly, the protocols differed between trials making it difficult to recommend a specific exercise programme. This may partly explain the significant heterogeneity in the included studies. However, despite the significant heterogeneity, the random effects meta-analysis suggests that the reduction in NP levels associated with exercise training is possible within 12 weeks. There remains limited mortality data in the trials of cardiac rehabilitation programmes which document NP level changes over time. It is thus impossible to determine whether a reduction in NP levels secondary to exercise therapy is associated with increased survival. It is possible that an exercise intervention may improve other patient reported outcomes, although these were not uniformly reported in the included trials. Finally, as all of the trials were not blinded to the patient or investigator, it is possible that there may be co-intervention bias associated with the exercise arm of these trials.

Our review differs from the two previous systematic reviews which have examined NP levels in non-surgical patients after exercise therapy (Pearson et al. 2018; Smart and Steele 2010). These reviews also found that NP levels were reduced after exercise therapy, with a high heterogeneity in the response. The strength of our review is that it updates the previous reviews (Smart and Steele 2010) with more trials and only includes RCTs with aerobic exercise programmes (Pearson et al. 2018). We did not consider trials examining yoga, stretching, Tai chi, functional electrical stimulation, or inspiratory muscle training (Pearson et al. 2018).

Elevated preoperative NP levels have been independently associated with major adverse cardiac events and mortality following surgery (Rodseth et al. 2008, 2011, 2014). Further investigation into the role of supervised preoperative exercise programme in the surgical population may provide insight into the relationship between exercise and NP levels in this cohort of patients.

## Conclusion

This meta-analysis shows that NP levels can be lowered with supervised exercise training and can be achieved within a 12-week programme. An exercise prehabilitation programme of 12 weeks duration may lower NP levels, and possibly perioperative risk. It is unclear whether this will improve postoperative cardiovascular outcomes.

## Supplementary information

**Additional file 1:** Example of search strategy for the systematic review.

**Additional file 2:** AMSTAR evaluation of previous systematic reviews.

**Additional file 3: Figure S1.** Risk of bias graph.

**Additional file 4: Figure S2.** Risk of bias summary.

## Data Availability

All articles available online and datasets are available from the corresponding author.

## Abbreviations

AT: Anaerobic threshold
BNP: B-type natriuretic peptide
CI: Confidence interval
EF: Ejection fraction
HR: Hazard ratio
IQR: Interquartile range
NP: Natriuretic peptide
NT-proBNP: N-terminal pro-B-type natriuretic peptide
SD: Standard deviation
SMD: Standardised mean difference
VO_2_: Maximum oxygen consumption
VT: Ventilatory threshold
