# Supplementary material for: The role of cardiac rehabilitation using exercise to decrease natriuretic peptide levels in non-surgical patients: a systematic review
Source: Perioper Med (Lond). 2019 Nov 18;8:14. doi: 10.1186/s13741-019-0124-0 (PMC6859626; doi:10.1186/s13741-019-0124-0)
Supplement: Supplementary file 1 — Additional file 1: Example of search strategy for the systematic review. [file 13741_2019_124_MOESM1_ESM.docx]

**Additional file 1. Example of search strategy for the systematic review**

Search strategy: (peptide set AND therapy set) NOT surgery set

Natriuretic Peptide, Brain (MeSH) OR

Brain Natriuretic Peptide OR Type-B Natriuretic Peptide OR Type B Natriuretic Peptide OR B-type Ventricular Natriuretic Peptide OR B type Ventricular Natriuretic Peptide

**AND**

Therapeutics (MeSH)

Therapeutics OR treatment OR therapy OR rehabilitation

**NOT**

General Surgery (MeSH) OR Surgical Procedures, Operative (MeSH) OR

surgery OR surgical OR operative

search strategy: (peptide set AND heart failure set) NOT surgery set

Natriuretic Peptide, Brain (MeSH) OR

Brain Natriuretic Peptide OR Type-B Natriuretic Peptide OR Type B Natriuretic Peptide OR B-type Ventricular Natriuretic Peptide OR B type Ventricular Natriuretic Peptide

**AND**

Heart Failure (MeSH) OR Coronary Artery Disease (MeSH)

Heart failure OR cardiac failure OR heart decompensation OR myocardial infarction OR myocardial ischemia OR heart attack OR myocardial infarct

**NOT**

General Surgery (MeSH) OR Surgical Procedures, Operative (MeSH) OR

surgery OR surgical OR operative

search strategy: (peptide set AND exercise set) NOT surgery set

Natriuretic Peptide, Brain (MeSH) OR

Brain Natriuretic Peptide OR Type-B Natriuretic Peptide OR Type B Natriuretic Peptide OR B-type Ventricular Natriuretic Peptide OR B type Ventricular Natriuretic Peptide

**AND**

Exercise (MeSH) OR Physical Exertion (MeSH) OR

Exercise OR physical exertion OR physical activity OR motor activity OR physical effort

**NOT**

General Surgery (MeSH) OR Surgical Procedures, Operative (MeSH) OR

surgery OR surgical OR operative
