# Supplementary material for: The role of cardiac rehabilitation using exercise to decrease natriuretic peptide levels in non-surgical patients: a systematic review
Source: Perioper Med (Lond). 2019 Nov 18;8:14. doi: 10.1186/s13741-019-0124-0 (PMC6859626; doi:10.1186/s13741-019-0124-0)
Supplement: Supplementary file 2 — Additional file 2: AMSTAR evaluation of previous systematic reviews. [file 13741_2019_124_MOESM2_ESM.docx]

**Additional file 2. AMSTAR evaluation of previous systematic reviews**

| Author | Journal | Comment | A piori design | Duplicate | Comprehensive review | Publication status | List of studies | Characteristics of  studies | Scientific quality assessed | Quality with conclusions | Publication bias discussed |
| --- | --- | --- | --- | --- | --- | --- | --- | --- | --- | --- | --- |
| Smart, Steele(Smart and Steele 2010) | International Journal of Cardiology  2009 | Systematic review and individual patient meta-analysis | No | Yes | No | No | Yes | Yes | Yes | Yes | No |
| Pearson, King, Smart(Pearson et al. 2018) | Open Heart 2018 | Systematic review and meta-analysis | No | Yes | Yes | No | Yes | Yes | Yes | Yes | Yes |
