# Supplementary figures and images for: The role of cardiac rehabilitation using exercise to decrease natriuretic peptide levels in non-surgical patients: a systematic review
Source: Perioper Med (Lond). 2019 Nov 18;8:14. doi: 10.1186/s13741-019-0124-0 (PMC6859626; doi:10.1186/s13741-019-0124-0)

**
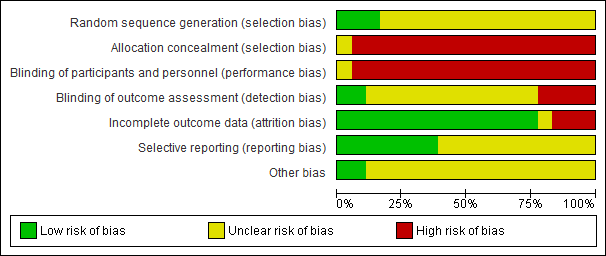
**

**Additional file 3: Figure S1. Risk of bias graph.**

Supplement: Supplementary file 3 — Additional file 3: Figure S1. Risk of bias graph. [file 13741_2019_124_MOESM3_ESM.docx]
